# Application of Duplex Fluorescence Melting Curve Analysis (FMCA) to Identify Canine Parvovirus Type 2 Variants

**DOI:** 10.3389/fmicb.2019.00419

**Published:** 2019-03-05

**Authors:** Zhicheng Liu, Gali Bingga, Chunhong Zhang, Junjie Shao, Haiyan Shen, Junying Sun, Jianfeng Zhang

**Affiliations:** ^1^Scientific Observation and Experiment Station of Veterinary Drugs and Diagnostic Techniques of Guangdong Province, Ministry of Agriculture, Key Laboratory of Livestock Disease Prevention of Guangdong Province, Institute of Animal Health, Guangdong Academy of Agricultural Sciences, Guangzhou, China; ^2^Vocational and Technical College of Inner Mongolia Agricultural University, Baotou, China; ^3^Changzhou Wumu Animal Hospital, Changzhou, China

**Keywords:** CPV-2 variant, genotyping, melting curve, TaqMan probe, sequencing

## Abstract

Canine parvovirus (CPV-2) is an enteric virus causing morbidity and mortality in dogs worldwide. Since CPV-2 emerged as canine pathogen, the original CPV-2 strain has constantly evolved, and its primary variants (CPV-2a, CPV-2b, and CPV-2c) co-circulate to varying extents in canine populations worldwide. Thus, rapid and accurate laboratory diagnoses of CPV-2 variants are crucial to monitor CPV-2 evolution. Conventional methods for CPV-2 genotyping are laborious, time consuming, and determining the genotype of a CPV-2 variant often requires two or more reaction tubes. The present study developed a probe-based fluorescence melting curve analysis (FMCA) for genotyping six different CPV-2 variants (original CPV-2, CPV-2a, CPV-2b, CPV-2c, and vaccine strains of CPVpf and CPVint) in a single reaction tube using only two TaqMan probes. One of the TaqMan probes (FAM labeled) was designed to perfectly match with the target sequence of CPV-2a, this probe allows a 1-bp mismatched hybridization with the CPV-2b VP2 gene region (A4062G), and a 2-bp mismatched hybridization for CPV-2c (A4062G and T4064A); Another TaqMan probe (HEX labeled) was produced to perfectly match with the target sequence of original CPV-2, this probe enables 1-bp mismatched hybridization with the other CPV-2 variants (A3045T). Using the two TaqMan probes, all six CPV-2 variants were readily distinguished by their respective melting temperature values in a single reaction tube. The detection limits of this assay were 1–10 copies per reaction for six CPV-2 construction plasmids and no cross reactions were observed with several other common canine viruses. In this assay, co-infected samples were also directly identified via probe-based FMCA without using a mixing control; only a pure control is required. The clinical evaluation of this assay was demonstrated by analyzing 83 clinical fecal samples, among which 41 (49.39%), 8 (9.63%), and 14 (16.87%) samples were found to be positive for CPV-2a, CPV-2b, and CPV-2c, respectively. The concordance rate between probe-based FMCA and Sanger sequencing was 100%. Thus, the duplex FMCA is effective, rapid, simple, high-throughput, and straightforward for genotyping CPV-2 variants, and is useful to effectively diagnose and monitor CPV-2 epidemiology.

## Introduction

Canine parvovirus type 2 (CPV-2), which was first identified in 1977–1978, causes acute gastroenteritis and leukopenia in dogs. A new antigenic variant (CPV-2a) completely replaced the originally identified CPV-2 virus between 1979 and 1980. Additional antigenic variants, CPV-2b and CPV-2c, were identified in 1984 (Parrish et al., 1991) and 2000 (Buonavoglia et al., 2001), respectively. Novel antigenic variants of CPV-2a/2b are still being identified, including new CPV-2a, new CPV-2b (Ohshima et al., 2008), Asp-300(2a), and Asp-300(2b) (Ikeda et al., 2000). Today, the CPV-2 variant originally identified in 1977 can only be found in some vaccine strains. The primary determinants of the CPV-2 genotype are codons 84, 87, 101, 297, 300, 425, and 426 in the VP2 protein (Decaro et al., 2006a,b,c; Yoon et al., 2009; Decaro and Buonavoglia, 2012; Bingga et al., 2014).

To control the disease, live modified vaccines have been widely used. Despite this, the CPV-2 infection remains a major problem for pets (Decaro and Buonavoglia, 2017). Furthermore, these live vaccines may replicate in the gastrointestinal tract and are shed along with the feces of vaccinated dogs (Gizzi et al., 2014), which has been suspected to interfere with diagnostic tests for clinical signs of acute gastroenteritis. As variants of CPV-2 emerged, the CPV-2 vaccine appeared to provide relatively lower and shorter immunity against heterologous CPVs (Decaro et al., 2008; Zhang et al., 2010). This observation can lead to a diagnostic dilemma when pups that have been administered with a CPV-2 vaccine recently present with clinical signs of acute gastroenteritis (Decaro et al., 2007). Based on these findings, CPV-2 variants need to be identified effectively for diagnosis and epidemiological monitoring.

Several approaches have been employed to type CPV-2 based on serological assays, such as the hemagglutinin inhibition (HI) test using monoclonal antibodies (Nakamura et al., 2003) and by molecular methods, like multiplex amplification refractory mutation system PCR (ARMS-PCR) (Chander et al., 2016), mini-sequencing based single-nucleotide polymorphism (SNP) analysis (Naidu et al., 2012; Pavana Jyothi et al., 2016), peptide nucleic acid-based (PNA) array (An et al., 2012), multiplexed tandem PCR (MT-PCR) (Meggiolaro et al., 2017), probe based minor binding groove assays (MBG assay) (Decaro et al., 2006a,b,c), high resolution melting curve analysis (HRM) and sequence analysis. However, HI testing needs at least four monoclonal antibodies (Nakamura et al., 2003) to identify CPV-2 variants, and most of the molecular methods need at least two reactions (Decaro et al., 2006a,b,c; An et al., 2012; Naidu et al., 2012; Bingga et al., 2014; Chander et al., 2016; Pavana Jyothi et al., 2016) or/and two technologies (An et al., 2012; Naidu et al., 2012; Bingga et al., 2014; Pavana Jyothi et al., 2016; Meggiolaro et al., 2017); Sequence analysis is regarded as the “gold standard” for CPV-2 genotyping, it is still time consuming, and labor intensive; In our previous study, PCR coupled with high resolution melting (HRM) curve analysis has been developed to genotype CPV-2 variants (Bingga et al., 2014). HRM is a simple, rapid, and inexpensive method for CPV-2 genotyping; however, it is not feasible for detecting multiple mutation sites of CPV-2 variants in a single test tube using DNA binding dye. In addition, due to the very small melting temperature difference (0.2°C), genotyping CPV-2 variants had to be performed by generating heteroduplexes in HRM analysis (Bingga et al., 2014). Probe-based fluorescence melting curve analysis (FMCA), however, could overcome those limitations mentioned above.

Probe-based FMCA is a powerful tool for SNP genotyping of target sequences based on the melting temperature generated by thermal denaturation of the probe-target hybrid (El Housni et al., 2003; Huang et al., 2011). With the probe-based FMCA method, the large range of melting temperature difference, 4–10°C, can be caused by only one or two SNPs and by using different fluorescence channels, it is possible to detect significantly greater mutation sites in one reaction tube (El Housni et al., 2003; Huang et al., 2011; Liu et al., 2018). Thus, to increase the discriminatory power comes from the melting temperature for genotyping CPV-2 variants, probe-based FMCA method was developed to detect the same mutant sequence in this study.

## Materials and Methods

### Strain Collection and DNA Extraction

A total of 113 fecal samples (30 CPV-2 fecal samples of known genotypes collected in 2013, and 83 field samples collected in 2017 and 2019), four batches of CPV-2 vaccines, and six plasmids (two as reference and four as control plasmids) were used in this study (Supplementary Table S1). The plasmid p-CPV-2 was synthesized based on the *VP2* gene of CPV-b (original CPV-2; GenBank accession no. M38245). Five plasmids of pCPV-2a, pCPV-2b, pCPV-2c, pCPVpf, and pCPVint were generated using the pMD18-T Vector Cloning Kit (Takara, Dalian, China), and the insert fragments of the plasmids were amplified from CPV-js1 (CPV-2a; GenBank accession no. KJ754512), CPV-js2 (CPV-2b; KJ754513), CPV-js4 (CPV-2c; KJ754515), CPVpf (Yoon et al., 2009) (CPVpf; isolated from commercial Vanguard^®^ Plus 5, Pfizer Inc., Lincoln, NE, United States), and CPVint (Yoon et al., 2009) (CPVint; isolated from commercial vaccine Nobivac^®^ DHPPi, Intervet Inc., Netherlands) using primer set CPV-1270F/1270R (Table 1), respectively.

**Table 1:** Primers and probes used in this study.

Primer/probe	Sequence (5′–3′)	Genome position^a^	Interpretation
87f-1	GAAATCACAGCAAACTCAAGC	2964–2984	Duplex FMCA assay
87r-jb	GCATGARTATCATCTAAAGCC	3094–3074	
cpv-F2	AGCACATCAAGATACAGGAAGATATCC	3989–4015	
ab-Rev	CCAATTGGATCTGTTGGTAGCAATACA	4096–4070	
P1	HEX-TGTAAATAATATGGATAAAACTGC-BHQ1	3035–3058	
P2	6-FAM-CCTTCCTGTAACAAATGATAATGTATT-BHQ1	4049–4075	
CPV-1270F	TGGAAATCACAGCAAACTC	2962–2980	Bingga et al. (2014), 1270 bp, generating plasmids, sequencing primers
CPV-1270R	AGTCTTGGTTTTAAGTCAGTATC	4231–4209	


*^a^Positions refer to the nucleotide sequences of strain CPV-b (accession no. M38245)*.

Viral RNA/DNA was extracted using the MiniBEST Viral RNA/DNA Extraction Kit version 4.0 (Takara, Dalian, China) according to the manufacturer’s instructions.

### Primer and Probe Design

**FIGURE 1:**
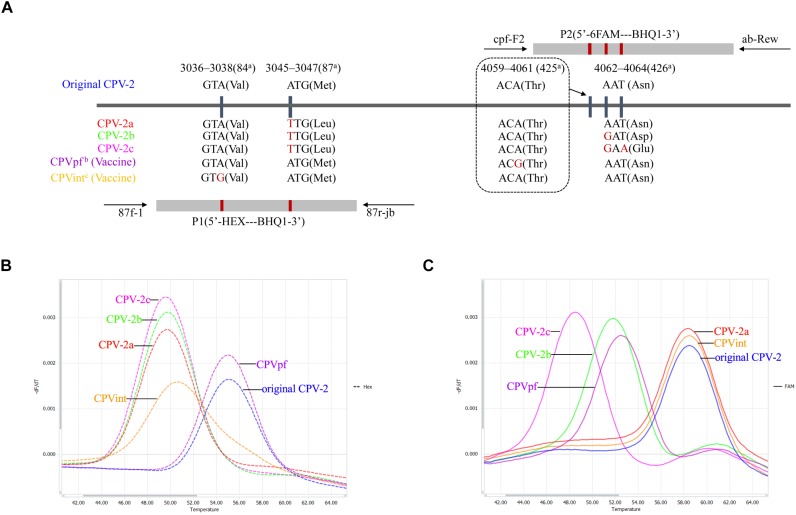
Schematic illustration of the duplex FMCA method. **(A)** Relative binding positions of probes along the CPV-2 genome. Melting peak calculation by derivative plotting of –dF/dT versus temperature in the HEX channel **(B)** and the FAM channel **(C)**. Blue, red, green, pink, purple, and orange curves represent original CPV-2, CPV-2a, CPV-2b, CPV-2c, and vaccine strains CPVpf and CPVint variants, respectively. ^a^Positions refer to the codons of VP2 capsid protein from strain CPV-b (accession no. M38245); ^b^CPVpf: accession no. FJ197847; ^c^CPVint: accession no. FJ197846.

Three sets of primers and two dual-labeled probes, designed to target the *VP2* gene of CPV-2, were used in this study (Table 1). The primer sets 87f-1/87r-jb, cpv-F2/ab-Rev, and probes P1 and P2, were designed to differentiate between the vaccine strains of CPVpf and CPVint, original CPV-2, CPV-2a, CPV-2b, and CPV-2c (Figure 1 and Table 1). P1 was designed to perfectly match with the target sequence of original CPV-2, while P2 was a perfect match with CPV-2a (Figure 1A). The primer set CPV-1270F/1270R was used to generate recombinant plasmids, which were used to determine the limit of detection (LOD) of the assay and co-infection analysis, and also used to sequence the *VP2* gene. All primer and probe sequences were analyzed using BLAST to confirm their specificity.

### Duplex Real-Time PCR and Melting Curve Analysis

The duplex FMCA assay was performed on a LightCycler^®^ 96 real-time system (Roche, Switzerland) in a 10-μL reaction containing 1× Taq Plus Master Mix (Vazyme, Nanjing, China), 0.02 μM of primer 87f-1, 0.03 μM of primer cpv-F2, 0.2 μM of primer 87r-jb, 0.3 μM of primer ab-Rev, 0.2 μM of probe P1, 0.3 μM of probe P2, and 1 μL of DNA template. Amplification started with denaturation for 5 min at 95°C, followed by amplification using 55 cycles of 95°C for 15 s, 55°C for 15 s, and 72°C for 15 s. Melting curve analysis was initiated with denaturation at 95°C for 180 s, followed by hybridization at 37°C for 180 s, and a stepwise temperature increment from 37 to 97°C at an ramp rate of 0.1°C/s, with a duration of 1 s. Fluorescence from both the FAM and HEX channels was measured at each step during the melting curve analysis. Melting curves were obtained by plotting the negative derivative of the fluorescence intensity with respect to temperature (-dF/dT) versus temperature (T). The *Tm* value for each probe was automatically obtained by identifying the peak of the corresponding melting curve. The melting *Tm* difference (Δ*Tm*) between reference plasmid p-CPV-2 and detected samples was calculated in the HEX channel, while the reference plasmid was pCPV-2a in the FAM channel.

### Sensitivity, Specificity, and Reproducibility

To study the analytical sensitivity of the duplex FMCA assay, six plasmids (p-CPV-2, pCPV-2a, pCPV-2b, pCPV-2c, pCPVpf, and pCPVint) were serially diluted 10-fold with water to concentrations of 1 × 10^9^ copies/μL to 1 × 10^0^ copies/μL, and the limit of detection for each genotype was determined from the lowest concentration measured by the duplex FMCA assay.

To evaluate the reproducibility of the assay, duplex FMCA was performed on different days using six plasmids and known genotypes of 30 CPV-2 fecal samples. Canine distemper virus (CDV), canine adenovirus type 2 (CAV-2), Canine Corona Virus (CCV), Canine rotavirus (CRV), canine parainfluenza virus (CPIV), and six plasmids were used to study the specificity of the duplex FMCA assay.

### Co-infection Analysis

To study the ability of the duplex FMCA assay to distinguish two genotypes when they are present simultaneously in the same sample, two of the six plasmids (p-CPV-2, pCPV-2a, pCPV-2b, pCPV-2c, pCPVpf, and pCPVint) were artificially polled at various ratios (10:0, 9:1, 8:2, 7:3, 6:4, 5:5, 4:6, 3:7, 2:8, 1:9, and 0:10) provided the overall template concentration was 10^8^ copies per reaction, were detected using the duplex FMCA assay.

### Clinical Study

A total of 83 field samples, assessed using an antigen test kit (Bio Note, South Korea) (Supplementary Table S1) were analyzed using the duplex FMCA assay, and confirmed using HRM and sequencing, as described previously (Bingga et al., 2014).

## Results

In this study, known genotypes of thirty CPV-2 fecal samples and two vaccine samples were used to establish the method. The results showed that the developed duplex FMCA assay using dual-labeled, self-quenching probes could identify original CPV-2, CPV-2a, CPV-2b, CPV-2c, and vaccine strains CPVpf and CPVint based on the *Tm* values and the type of fluorescence of the melting peaks, as shown in Figure 1B,C. For each sample, two *Tm* values were obtained from the HEX and FAM channels, corresponding to probe P1 and P2, respectively. In the HEX channel, strains CPV-2a, CPV-2b, and CPV-2c were clustered together with *Tm* values of 50.01 ± 0.26°C, 49.91 ± 0.29°C, and 49.82 ± 0.19°C, respectively; the original CPV-2 and CPVpf strains were clustered together with *Tm* values of 55.10 ± 0.04°C and 55.17 ± 0.25°C, respectively. These two clusters were easily differentiated from each other by their *Tm* values. In this channel, the vaccine strain of CPVint was also readily distinguished from other strains based on the broader melting peak shape (Figure 1B). In the FAM channel, strains CPV-2a, CPV-2b, and CPV-2c were separate from each other, with the *Tm* values of 58.37 ± 0.30°C, 51.87 ± 0.31°C, and 48.85 ± 0.36°C, respectively. The original CPV-2 and CPVpf strain were also separate from each other, with the *Tm* values of 58.42 ± 0.07°C and 52.47 ± 0.33°C, respectively. In this channel, although the vaccine strain of CPVpf yielded a *Tm* value of 52.47 ± 0.33°C, which was only 0.60°C higher than that of CPV-2b, the *Tm* difference between them was 5.16°C in the HEX channel. Thus, it was obviously feasible to distinguish all of the different CPV strains based on *Tm* differences and melting curve shapes in the HEX and FAM channels.

### Sensitivity, Specificity, and Reproducibility

Serial dilutions of the six recombinant plasmids, p-CPV-2, pCPV-2a, pCPV-2b, pCPV-2c, pCPVpf, and pCPVint, ranging from 1 × 10^9^ to 1 × 10^0^ copies/μL, were tested using the duplex FMCA assay, and fluorescence signals corresponding to melting peaks were obtained between 1 × 10^0^ and 1 × 10^9^ copies per reaction for each plasmid except p-CPV-2 (1 × 10^1^ and 1 × 10^9^ copies per reaction) (Supplementary Figure S1). Therefore, the detection limit of the duplex FMCA assay was determined as 1 × 10^0^ copies per reaction for recombinant plasmids pCPV-2a, pCPV-2b, pCPV-2c, pCPVpf, and pCPVint, and 1 × 10^1^ copies per reaction for p-CPV-2.

The specificity of duplex FMCA was validated using DNA or cDNA (complementary DNA) samples from six CPV-2 variants and five other viruses (Supplementary Figure S2). No specific melting peak was detected with the other non-targeted dog viruses such as CDV, CAV-2, CCV, CRV, and CPIV in either the FAM or HEX channels. These results suggested that the designed primer sets and probes were highly selective and specific for their target viruses, exhibiting no cross-reactivity with several other common canine viruses.

We next evaluated the run-to-run reproducibility of the duplex FMCA assay. Considering the narrow *Tm* windows, the assay was run on different days using known genotypes of 30 CPV-2 fecal samples and positive plasmids p-CPV-2, pCPVpf, and pCPVint. The result showed that the SD absolute values of *Tm* for all the tested samples determined from both probes ranged from 0 to 0.36°C (Table 2), which are lower than 1°C. This result demonstrated that the discriminatory power of the developed method could be ensured by the high reproducibility of the *Tm* values.

**Table 2:** Reproducibility testing of bicolor FMCA assay for the known genotypes of thirty CPV-2 fecal samples and two vaccine samples.

Genotype	No. of times tested	P1 (HEX channel)		P2 (FAM channel)	
		^c^Tm, °C (Mean ± SD)	^a^ΔTm, °C (Mean ± SD)	^d^Tm, °C (Mean ± SD)	^b^ΔTm, °C (Mean ± SD)
Original CPV-2	10	55.10 ± 0.04^a^	0.03 ± 0.03	58.42 ± 0.07^a^	0.08 ± 0.04
CPV-2a	186	50.01 ± 0.26^b^	5.35 ± 0.18	58.37 ± 0.30^a^	0.37 ± 0.20
CPV-2b	72	49.91 ± 0.29^b^	5.45 ± 0.20	51.87 ± 0.31^b^	6.85 ± 0.22
CPV-2c	24	49.82 ± 0.19^b^	5.53 ± 0.10	48.85 ± 0.36^c^	9.86 ± 0.29
CPVpf	6	55.17 ± 0.25^a^	0.36 ± 0.12	52.47 ± 0.33^d^	6.25 ± 0.24
CPVint	6	50.55 ± 0.22^c^	4.80 ± 0.07	58.26 ± 0.22^a^	0.53 ± 0.10


*^a^ΔTm = | Tm (reference control of original CPV-2) – Tm (other)|^*b*^ΔTm = | Tm (reference control of CPV-2a) – Tm (other)|^*c*^Tm: Different letters indicate statistically significant differences (*P* < 0.05). ^*d*^Tm: Different letters indicate statistically significant differences (*P* < 0.05)*.

### Co-infection Analysis

To assess the capacity of FMCA to detect co-infection, unequal mixtures of two differently constructed plasmids were mixed at ratios ranging from 1:1 to 1:9 (v/v). By varying two of six plasmids relative ratios, the results showed that as low as 10% (1:9) of one genotype in the presence of 90% (9:1) of the other genotype was distinguishable in the mixed sample at a template concentration of 10^8^ copies per reaction. The relative height of the melting peaks was positively correlated with the ratio of the mixed genotypes (Supplementary Figures S3, S4). Considering that it is almost impossible for the two kinds of vaccines to be present in the same field sample, the assay was not evaluated when pCPVpf and pCPVint were simultaneously present in the same reaction.

**FIGURE 2:**
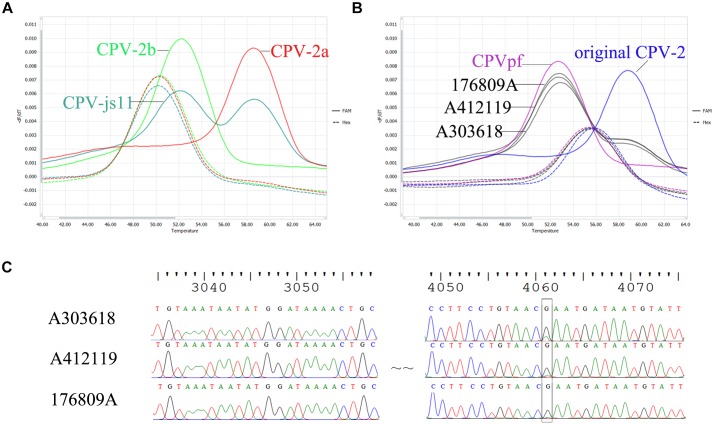
Comparison of sequencing chromatograms and melting peaks of co-infection. **(A)** Melting peaks generated by bicolor FMCA assay of the field sample CPV-js11 and **(B)** three batches of Vanguard^®^ Plus 5 vaccine. **(C)** Sequencing chromatograms of three batches of Vanguard^®^ Plus 5 vaccines.

To evaluate the differentiation power of the probe-based FMCA in co-infected field samples, strain CPV-js11, which had been identified to contain CPV-2a and CPV-2b using the HRM assay and DNA sequencing in previous study (Bingga et al., 2014) was detected using probe-based FMCA. The result showed that the co-infected sample CPV-js11 could be easily identified using the probe-based FMCA assay (Figure 2A).

In the present study, three batches of Vanguard^®^ Plus 5 vaccines (Batch No: A303618, A411088 and A412119) and one batch of Nobivac^®^ DHPPi vaccine (Batch No. B849A02) were also detected using the duplex FMCA assay. The results showed that the batch of Nobivac^®^ DHPPi vaccine contained only one strain, CPVint. However, all three batches of Vanguard^®^ Plus 5 vaccines included original CPV-2 and CPVpf strains, simultaneously (Figure 2B). To confirm the result, DNA sequencing (Bingga et al., 2014) was carried out to detect the strains in present in three batches of Vanguard^®^ Plus 5 vaccines. The sequencing results showed that Vanguard^®^ Plus 5 included a sleeve peak at nt 4061 of the complete CPV genome in the sequencing chromatography, which indicated the vaccine contained the original CPV-2 and CPVpf strains simultaneously (Figure 2C). For additional confirmation of the results, the purified PCR product from three batches of Vanguard^®^ Plus 5 were also cloned using a pMD18-T kit (Takara, Dalian, China), and two/three/three original CPV-2 and eight/seven/seven CPVpf were detected in ten random clones, respectively.

### Clinical Test

To estimate the capability of the probe-based FMCA method to distinguish field CPV-2 variants, 83 fecal samples collected in 2017 and 2019 were tested using the probe-based FMCA method (Supplementary Figure S5). Of the 83 fecal samples, 41 (49.39%) were positive for CPV-2a, 8 (9.63%) were positive for CPV-2b, 14 (16.87%) were positive for CPV-2c, and the remaining 20 samples were negative for CPV-2 (Supplementary Table S2). DNA sequencing of the fecal samples confirmed that the novel developed method could distinguish different CPV-2 strains at 100% accuracy (Supplementary Table S3).

## Discussion

Melting curve analysis in conjunction with real-time PCR, including high-resolution melting curve analysis (HRM) (Montgomery et al., 2007; Er and Chang, 2012; Bruzzone and Steer, 2015) and TaqMan probe-based FMCA (El Housni et al., 2003), has been widely used in clinical laboratories to detect SNPs. Compared with TaqMan probe-based FMCA, HRM is a simple, rapid, and inexpensive method for SNP testing. In our laboratory, PCR coupled with HRM curve analysis has also been developed to genotype Canine parvovirus type 2 (CPV-2) variants (Bingga et al., 2014). However, HRM analysis encounters technical difficulties when different CPV-2 genotypes from one sample need to be detected simultaneously in a single reaction tube. This problem can be solved using probe-based FMCA. The advantage of probe-based FMCA is its ability for multiplex detection using probe with specific melting temperatures. When the template DNA fully matches with the probe sequence, the probe will bind at the specific melting temperature. If the template DNA has a mismatch with the probe, the melting temperature will decrease remarkably compared with the matched hybrids. Thus, the *Tm* difference (Δ*Tm*) between the fully matched hybrid and the mismatched hybrids allows researchers to detect SNP variations in a single reaction tube.

In this study, two TaqMan probes were designed to identify six different CPV-2 variants using melting temperature difference in a single tube. A TaqMan probe is a typical dual labeled hydrolysis probe. A standard TaqMan probe consist of an 18–25 bp oligonucleotide probe that is labeled with a reporter fluorophore at its 5′ end and a quencher fluorophore at its 3′ end (Livak et al., 1995). In solution, the randomly coiled conformation enables fluorescence quenching because the quencher and the fluorophore remain in proximity to each other. Thus, a non-hybridized TaqMan probe is only weakly fluorescent. When the probe hybridizes with its target, strong fluorescence occurs because the quencher and fluorophore are separated by the length of the probe. After denaturation from the probe-target hybrid, the probe returns to its weakly fluorescent, randomly coiled conformation.

In this study, a FAM-labeled TaqMan probe was established that perfectly matched with the target sequence of CPV-2a. This probe allows a 1-bp mismatched hybridization with the CPV-2b VP2 gene region (A4062G), and a 2-bp mismatched hybridization with CPV-2c (A4062G and T4064A). The result of FMCA showed that the melting temperature differences (Δ*Tms*) between the fully matched hybrid and the mismatched hybrids were 6.85 ± 0.22°C and 9.86 ± 0.29°C for 1- and 2-bp mismatched hybridization, respectively. Therefore, the Δ*Tm* values were sufficient to classify the three melting temperatures clearly in a single detection experiment for CPV-2a, CPV-2b, and CPV-2c. However, one limitation was that using only the FAM-labeled probe, the assay could not discriminate CPV-2a from the original CPV-2, as both strains have similar nucleic acids at the target sequence (Figure 1A). Thus, a HEX-labeled TaqMan probe was produced to distinguish the original CPV-2 from CPV-2a strains, based on the melting temperature difference. The HEX-labeled TaqMan probe was designed to perfectly match with the target sequence of original CPV-2, in which other strain of CPV-2 variants have a 1-bp mismatch (A3045T). This mismatched hybridization resulted in a Δ*Tm* of 5.35 ± 0.18°C between original CPV-2 and CPV-2a. In addition, two attenuated live vaccine strains (CPVpf and CPVint) were also successfully identified using the two TaqMan probes, because of mutations A4061G and A3038G in the corresponding target sequences (Figure 1).

Notably, mismatched hybridization can cause a change in the melting temperature; however, the melting temperature can also been affected by a mismatched position in the TaqMan probe. In this study, strain CPV-2b and vaccine strain CPVpf both had a 1-bp mismatched hybridization with the FAM-labeled TaqMan probe, and only at the mismatched position was they different to each other. The values of the probe melting temperatures, however, were 51.87 ± 0.31°C and 52.47 ± 0.33°C for CPV-2b and CPVpf, respectively (*P* < 0.05). The same phenomenon also occurred on the HEX-labeled probe, with probe melting temperatures of 49.97 ± 0.27°C and 50.55 ± 0.22°C for CPV-2a/2b/2c and CPVint, respectively (*P* < 0.05). This may be caused by the different nearest-neighbor bases next to the mismatched site affecting the *Tm* of the probe (SantaLucia, 1998).

In our assay, the sequencing analysis was executed in parallel for all the field samples and the results showed that 100% accuracy was achieved from the probe-based FMCA method, while 98.23% accuracy was achieved for the HRM assay (Supplementary Table S3). The increased accuracy of FMCA derived from larger *Tm* differences than those in HRM between the different genotypes of CPV-2. For example, the melting temperature shifts between CPV-2b and CPV-2c were 3.2°C using probe-based FMCA, which was significantly higher than the *Tm* difference (0.2°C) between them in the HRM analysis in our previous study. In the HRM analysis, CPV-2b and CPV-2c were discriminated from each other by an additional hybridization step to generate heteroduplexes (Bingga et al., 2014). Moreover, distinguishing all the CPV genotypes, including two vaccine strains, had to be accomplished using four separate reaction tubes in the HRM assay (Bingga et al., 2014), while it was done in a single reaction tube with two labeled probes in FMCA.

Previously, co-infection with different CPV-2 variants has been reported sporadically in dogs, e.g., co-infections in clinical cases with CPV-2a and CPV-2c (Battilani et al., 2007; Perez et al., 2014), CPV-2a and CPV-2b (Bingga et al., 2014), and CPV-2 (vaccines) and CPV-2a (Decaro et al., 2007). In our laboratory, co-infection sample CPV-js11, containing CPV-2a and CPV-2b, was detected by HRM analysis in a previous study (Bingga et al., 2014). In the present study, two strains from the mixed sample of CPV-js11 were readily distinguished using the probe-based FMCA method. In comparison with the HRM method, the co-infected sample was directly identified by probe-based FMCA without using a mixing control, only a pure control is required (Figure 2). This is because the mixed sample contains both heteroduplex and homoduplex species after PCR, and the melting curve of the co-infected sample is a combination of their melting profiles (Montgomery et al., 2007). In the HRM assay, the higher melting temperature peak was a combination of two homoduplex melting profiles, and the lower melting temperature peak was also a combination of two heteroduplex melting profiles. In the probe-based FMCA, however, the higher melting temperature peak was derived from one duplex melting profile, which was perfectly matched with the probe; the lower melting temperature peak was derived from one duplex melting profile, which was mismatched with the probe. Thus, the components of the mixed sample were detected by the labeled probes directly from the four duplexes in the probe-based FMCA assay; however, it is not feasible to identify the ingredients in the co-infected sample using the intercalating dyes-based HRM assay without using a mixing control.

It is notable that when the developed probe-based FMCA assay was used to detect the commercial attenuated live vaccine of Vanguard^®^ Plus 5, two melting peaks were observed in the FAM channel. According to the *Tm* values, the vaccine contains CPVpf (52.27 ± 0.31°C) and original CPV-2 (58.32 ± 0.40°C) simultaneously, and the height of melting peak indicated that CPVpf is dominant. The sequencing results of the PCR products and cloning experiments both confirmed two different strains of CPVpf and original CPV-2 co-existed in the CPV-2 commercial vaccine Vanguard^®^ Plus 5. Thus, this developed method can also be used as a tool for rapid identification of vaccine purity.

It should be pointed out that the probe-based (bicolor) FMCA assay developed in this study was designed according to SNPs in codons 84, 87, 425, and 426, which were able to identify dominant types of CPV-2, including original CPV-2, CPV-2a, CPV-2b, CPV-2c, and vaccine strains of CPVpf and CPVint (Figure 1A). While, other codons (like 297 and 300) determine the antigenic variants of new CPV-2a/2b and Asp-300(2a/2b) (Decaro et al., 2006c; Decaro and Buonavoglia, 2012). Due to the classical CPV-2a/2b variants were almost replaced by new CPV-2a/2b and the sporadic cases of Asp-300(2a/2b) were occurred (Supplementary Figure S6), we have not developed a probe-based FMCA to differentiate new CPV-2a/2b variants from other CPV-2a/2b types. However, once the classical CPV-2a/2b or/and Asp-300(2a/2b) are prevalent again, a tricolor probe-based FMCA, based on codons 84, 87, 297, 300, 425, and 426, can be developed to solve this problem.

## Conclusion

In conclusion, this study describes, for the first time, a simple, rapid, accurate, high-throughput, and straightforward method to genotype CPV-2 strains, including original CPV-2, CPV-2a, CPV-2b, CPV-2c, and vaccine strains CPVpf and CPVint, by using duplex probe-based FMCA. The developed method was also useful to detect co-infected samples directly, without sequencing.

## Data Availability

The datasets generated for this study can be found in NCBI, MK076889–MK076943, and MK460553–MK460560.

## Ethics Statement

Ethics approval was not needed for this study from the Committee on the Ethics of Animal Experiments of Institute of Animal Health, Guangdong Academy of Agricultural Sciences, according to the Constitution on the Ethics of Animal Experiments of Institute of Animal Health, Guangdong Academy of Agricultural Sciences [(2016)15], and the guidelines of our institution.

## Author Contributions

ZL and GB conceived the study and wrote the manuscript. CZ carried out the data analysis. JjS collected and detected the field samples using an antigen test kit. HS and JyS participated in manuscript preparation. ZL, GB, CZ, and JZ designed the experiments. All authors read and approved the final manuscript.

## Conflict of Interest Statement

The authors declare that the research was conducted in the absence of any commercial or financial relationships that could be construed as a potential conflict of interest.
