# Supplementary material for: Application of Duplex Fluorescence Melting Curve Analysis (FMCA) to Identify Canine Parvovirus Type 2 Variants
Source: Front Microbiol. 2019 Mar 5;10:419. doi: 10.3389/fmicb.2019.00419 (PMC6411689; doi:10.3389/fmicb.2019.00419)
Supplement: Supplementary file 1 [file Data_Sheet_1.doc]

*Supplementary Material*

**Application of duplex fluorescence melting curve analysis (FMCA) to identify canine parvovirus type 2 variants**

**Zhicheng Liu^1^, ^†^ , Gali Bingga^2^, ^†^ , Chunhong Zhang^1, †^ , Junjie Shao^3^ , Haiyan Shen^1^ , Junying Sun^1^ and Jianfeng Zhang^1,*^**

^1^ Scientific Observation and Experiment Station of Veterinary Drugs and Diagnostic Techniques of Guangdong Province, Ministry of Agriculture, P.R.China, Key Laboratory of Livestock Disease Prevention of Guangdong Province, Institute of Animal Health, Guangdong Academy of Agricultural Sciences, Guangzhou, China, ^2^ Vocational and Technical College of Inner Mongolia Agricultural University, Baotou, China, ^3^ Changzhou Wumu Animal Hospital, Changzhou, China

^†^ These authors contributed equally to this work and share the first authorship.

* Correspondence: Jianfeng Zhang. E-mail: [13668939298@139.com](mailto:13668939298@139.com).

**Supplementary Figures and Tables**


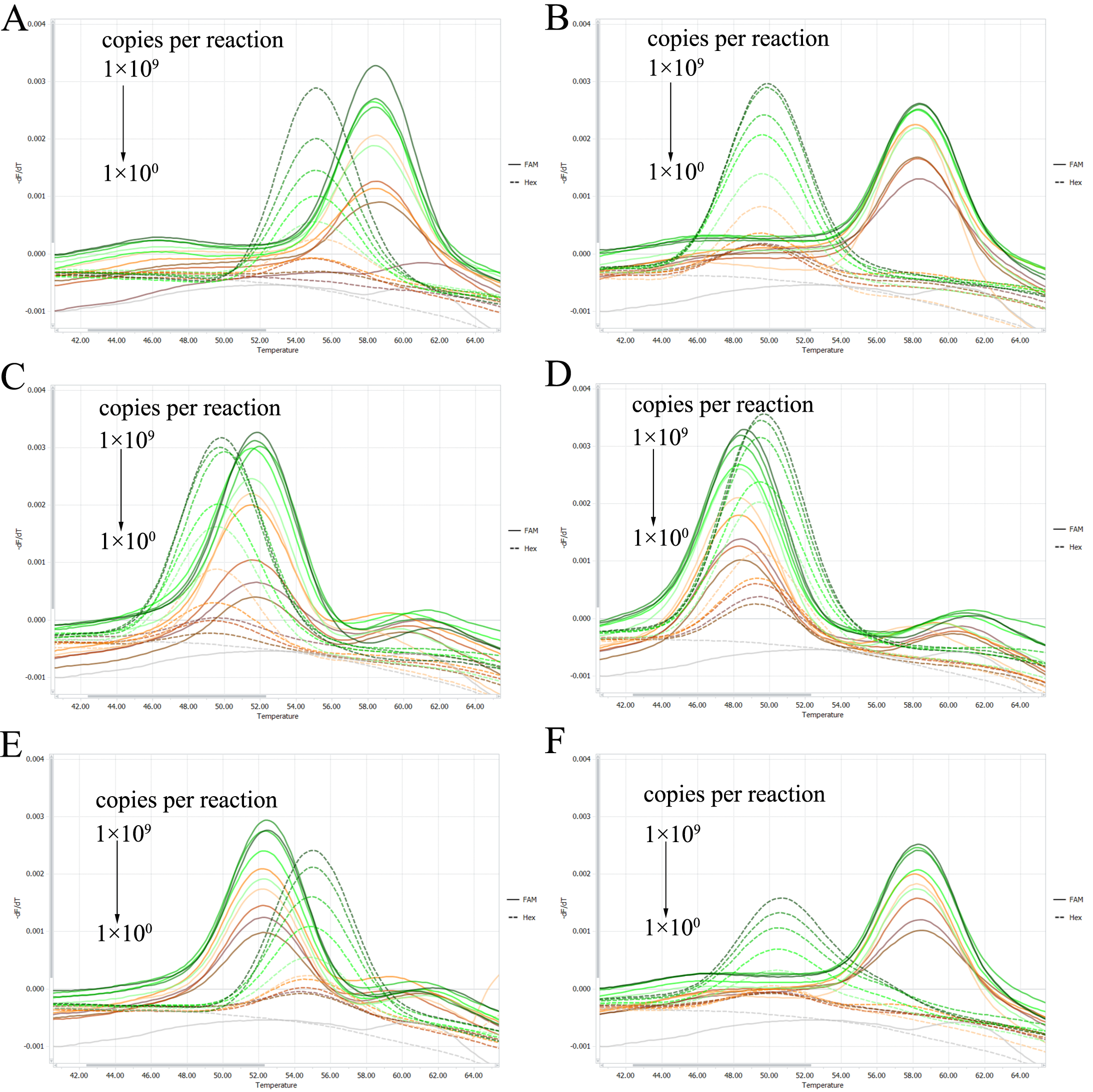


**Supplementary Figure S1. Sensitivity of the duplex FMCA method.** Melting curves from the duplex FMCA assay with reference recombinant plasmids (**A**) pCPV-2, (**B**) pCPV-2a, (**C**) pCPV-2b, (**D**) pCPV-2c, (**E**) pCPVpf, and (**F**) pCPVint, in the HEX (broken curve) and FAM (solid curve) channels, ranging from 1 × 10^0^ to 1 × 10^9^ copies per reaction (from bottom to top). The detection limits of the assay were 1.0 × 10^1^ copies per reaction for pCPV-2 and 1.0 × 10^0^ copies per reaction for other variant plasmids.


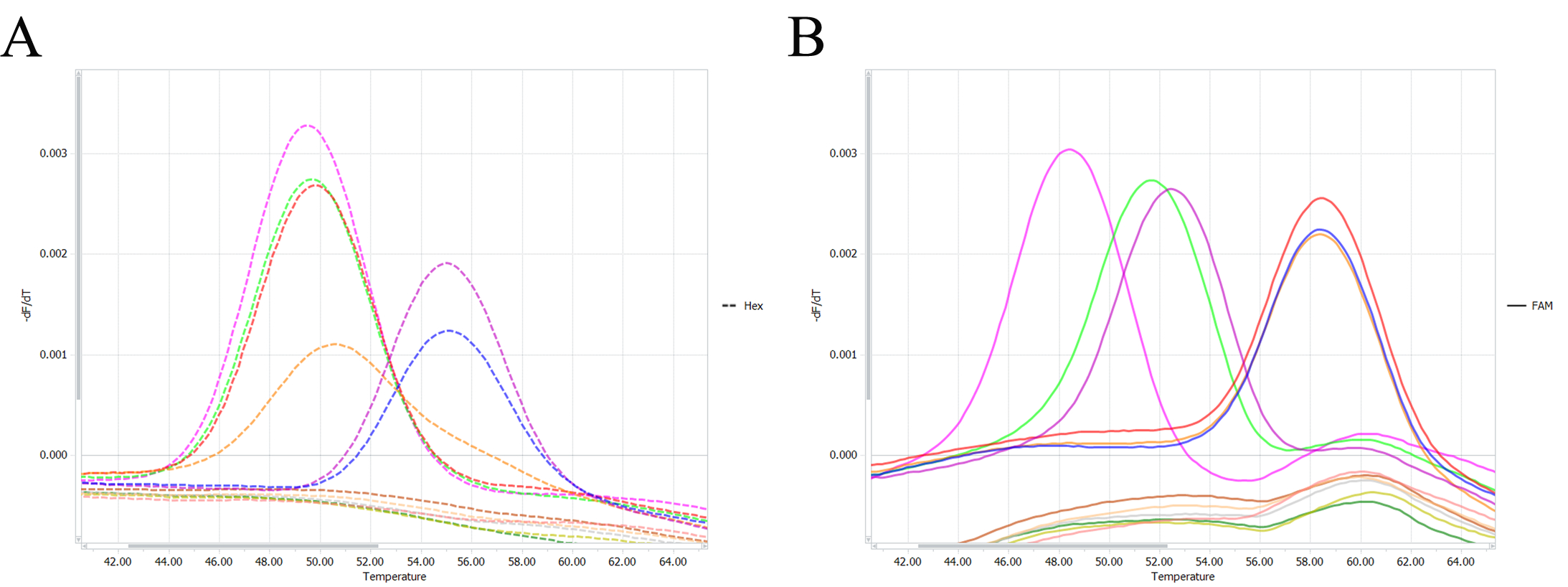


**Supplementary Figure S2. Specificity of the duplex FMCA method.** Melting peaks obtained from the duplex FMCA assay in the HEX channel (**A**, broken curve) and the FAM channel (**B**, solid curve) with six positive samples and five several common canine viruses. Reference recombinant plasmids pCPV-2, pCPV-2a, pCPV-2b, pCPV-2c, pCPVpf, and pCPVint served as positive controls, corresponding to the blue, red, green, pink, purple and orange curves, respectively, and a No Template Control (NTC) served as the negative control (NC, grey curve).


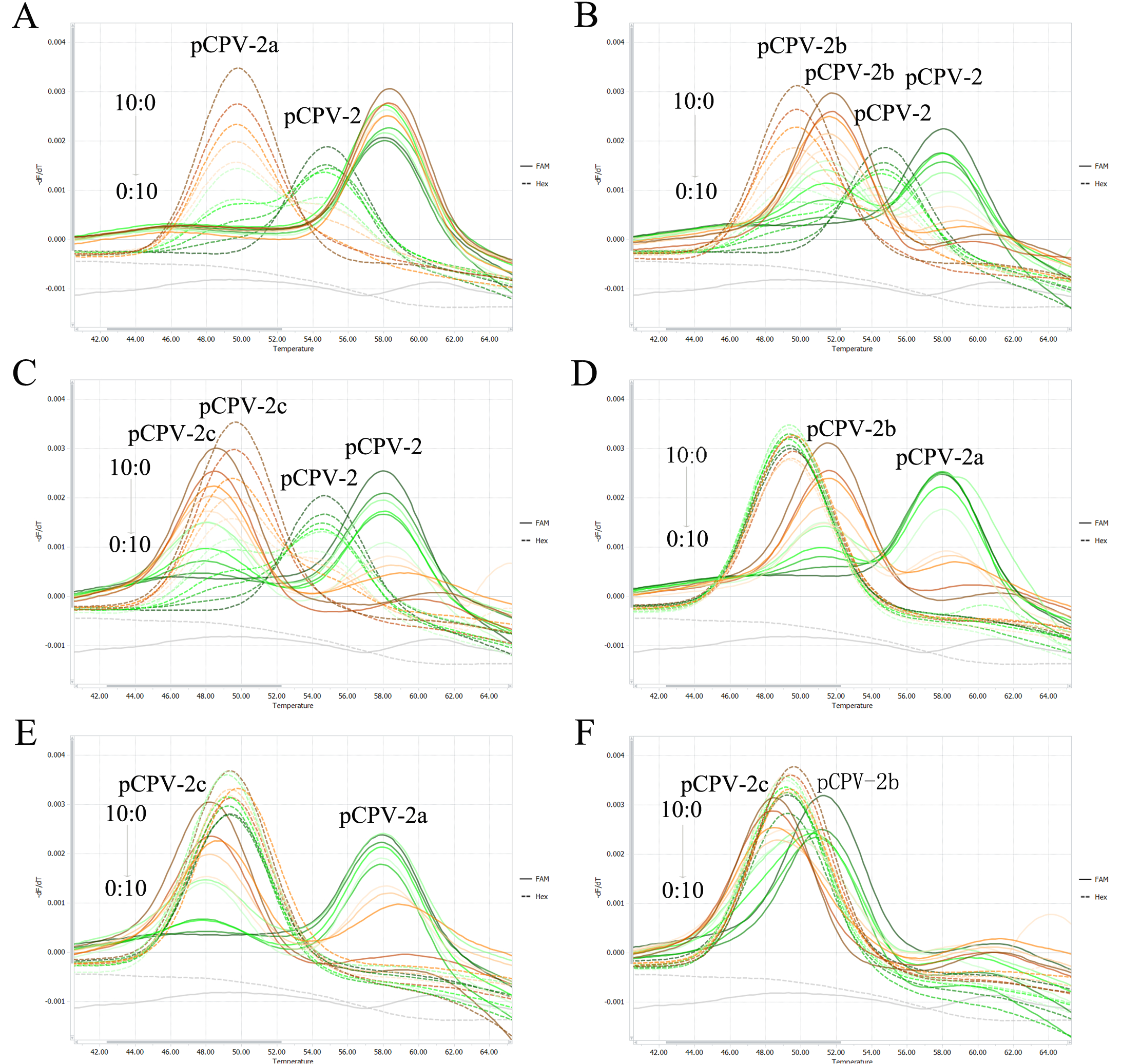


**Supplementary Figure S3. Simultaneous detection of field CPV-2 variants in mixed infections by the duplex FMCA method.** Two co-existing genotypes were detected in the HEX (broken curve) and FAM (solid curve) channels. Melting peaks of artificial plasmid templates containing pCPV-2 and pCPV-2a (**A**), pCPV-2 and pCPV-2b (**B**), pCPV-2 and pCPV-2c (**C**), pCPV-2a and pCPV-2b (**D**), pCPV-2a and pCPV-2c (**E**), pCPV-2b and pCPV-2c, and (**F**), at various ratios (10:0, 9:1, 8:2, 7:3, 6:4, 5:5, 4:6, 3:7, 2:8, 1:9, and 0:10) were tested. The overall template concentration was 10^8^ copies per reaction. The NTC negative control is represented by a grey curve.

**
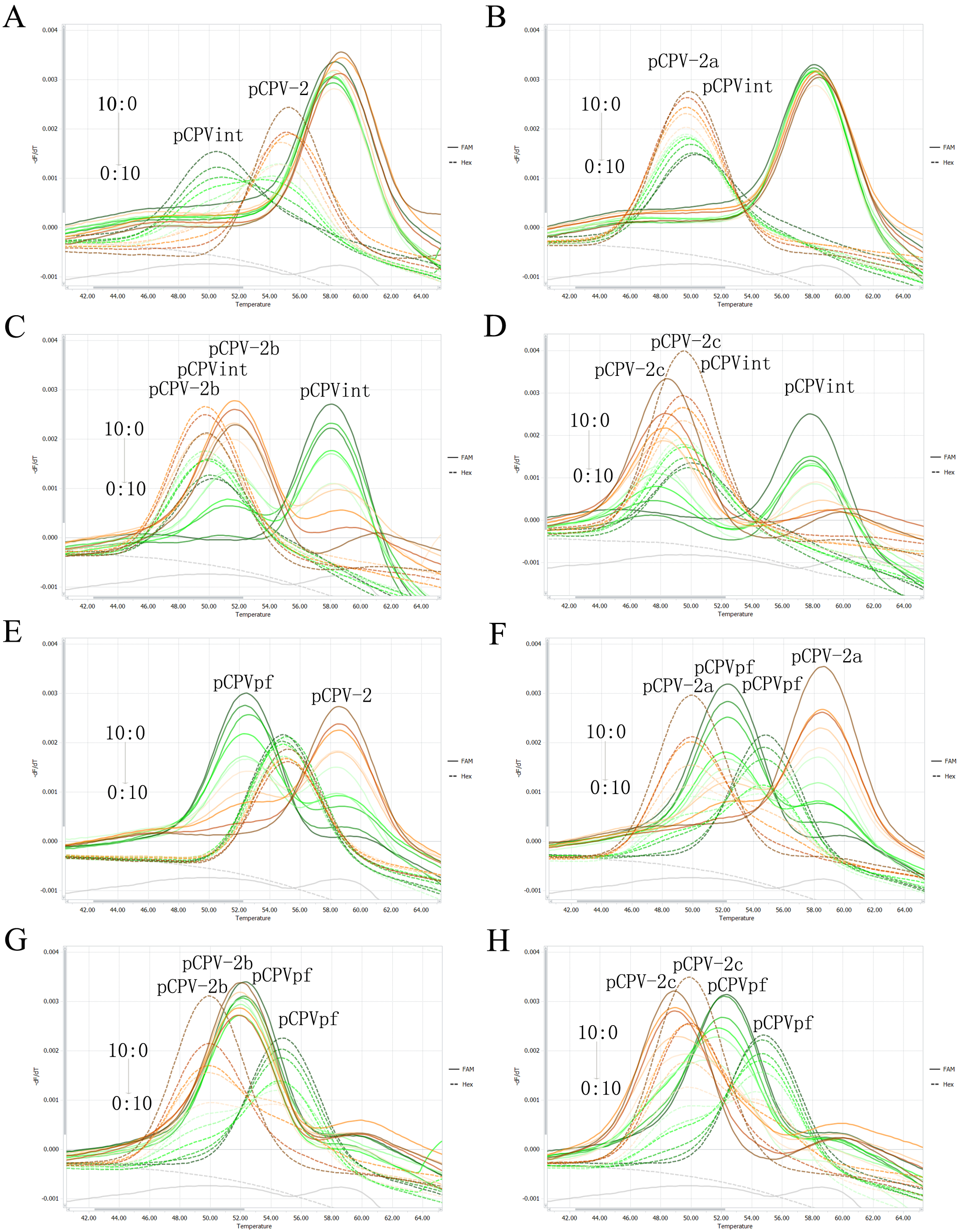
**

**Supplementary Figure S4. Simultaneous detection of original CPV-2 vaccines and field CPV-2 variants in mixed infections using the duplex FMCA method.** Two co-existing genotypes were detected in the HEX (broken curve) and FAM (solid curve) channels. Melting peaks of artificial plasmid templates containing pCPVint and pCPV-2 (**A**), pCPVint and pCPV-2a (**B**), pCPVint and pCPV-2b (**C**), pCPVint and pCPV-2c (**D**), pCPVpf and pCPV-2 (**E**), pCPVpf and pCPV-2a (**F**), pCPVpf and pCPV-2b (**G**), and pCPVpf and pCPV-2c (**H**), at various ratios (10:0, 9:1, 8:2, 7:3, 6:4, 5:5, 4:6, 3:7, 2:8, 1:9, and 0:10) were tested. The overall template concentration was 10^8^ copies per reaction. The NTC negative control is represented by a grey curve.


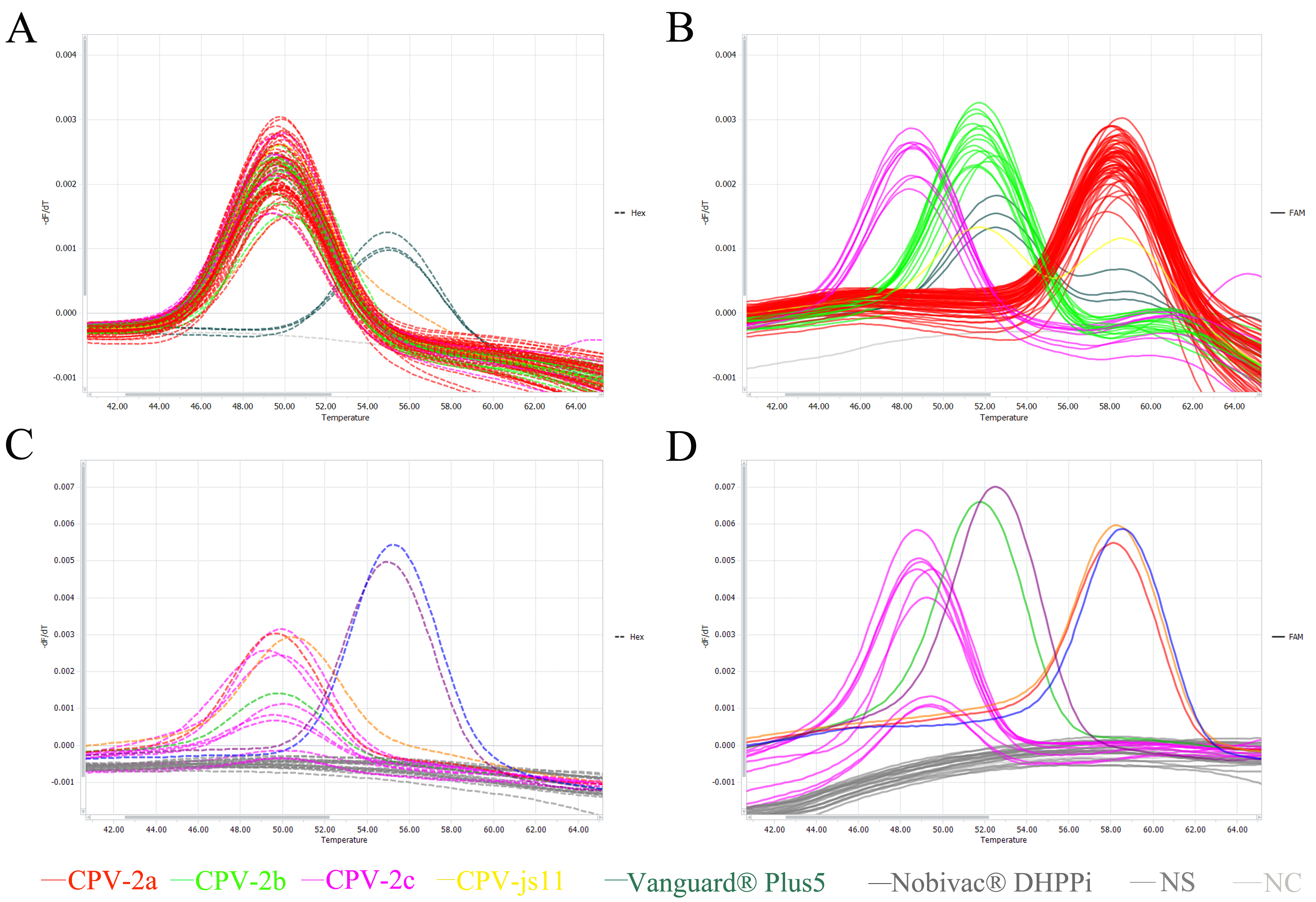


**Supplementary Figure S5. Melting peaks of clinical specimens and four CPV-2 vaccines generated by the duplex FMCA method.** Melting peaks obtained from duplex FMCA assay in the HEX channel (**A**, **C**, broken curve) and the FAM channel (**B**, **D**, solid curve). The NS were negtive samples, and the NC was negtive control.

**
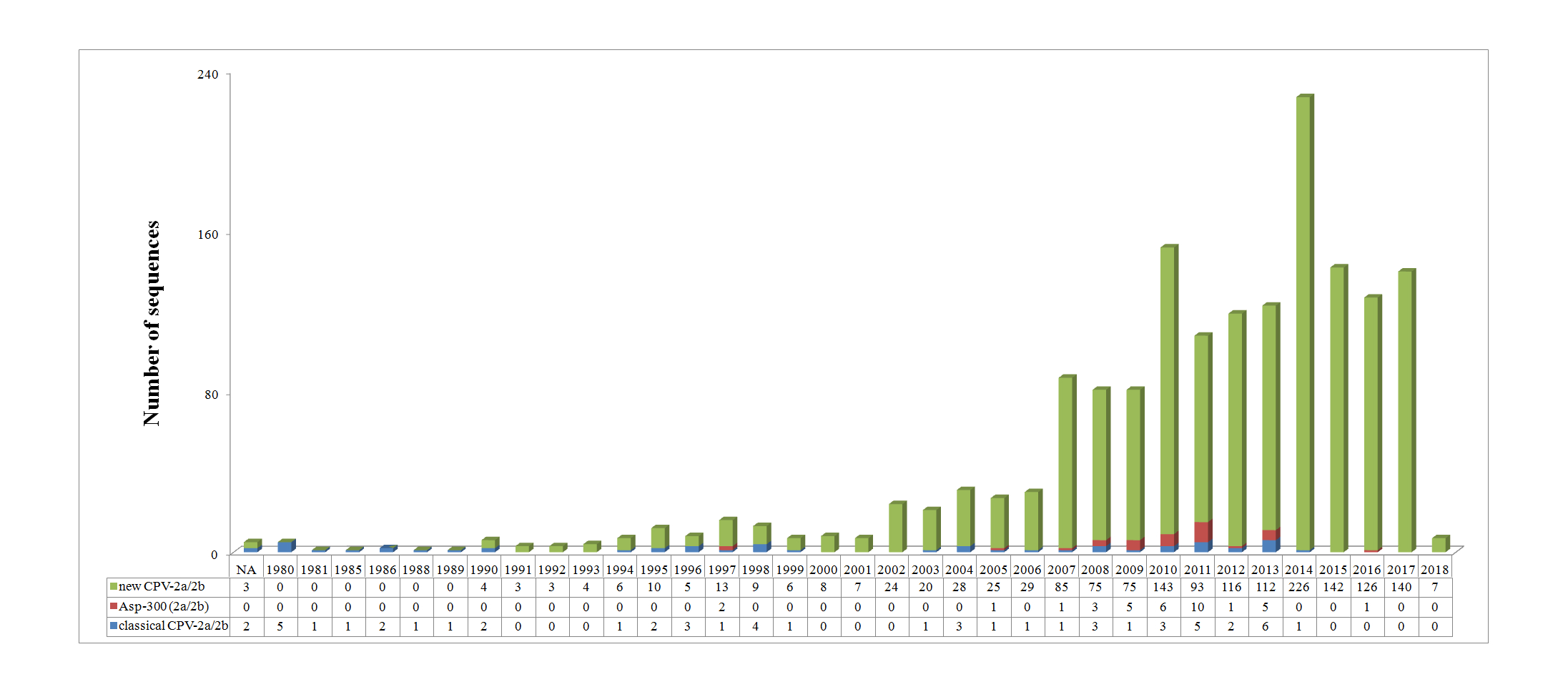
**

**Supplementary Figure S6. The prevalence of CPV-2a/2b variants.** Up to now, a total of 1630 CPV-2a/2b sequences (including classical CPV-2a, classical CPV-2b, new CPV-2a, new CPV-2b, Asp-300(2a) and Asp-300(2b)), were available for sequence analysis in GenBank (http://www.ncbi.nlm.nih.gov/genbank/). The result of sequence alignment showed that only 54 classical CPV-2a/2b (3.31%) were prevalent before 2015 and one in 2014; 33 Asp-300 (2a/2b) (2.02%) were prevalent from 2005 to 2013 and only two and one in 1997 and 2016, respectively. Thus, the current prevalent CPV-2a/2b variants were new CPV-2a/2b.

**Supplementary Table S1. CPV strains used in this study and their origin**

| **Sample Number** | **Sample ID** | **GenBank Accession Number** | **Geographical origin** | **Collection Year** | **GICT^a^** | **Genotype** | **Interpretation^b^** |
| --- | --- | --- | --- | --- | --- | --- | --- |
| **1** | pCPV-2 | — | — | — | — | Original CPV-2 | Control /Plasmid |
| **2** | pCPV-2a | — | — | — | — | CPV-2a | Control /Plasmid |
| **3** | pCPV-2b | — | — | — | — | CPV-2b | Control/ Plasmid |
| **4** | pCPV-2c | — | — | — | — | CPV-2c | Control/ Plasmid |
| **5** | pCPVpf | — | — | — | — | CPVpf | Control/ Plasmid |
| **6** | pCPVint | — | — | — | — | CPVint | Control/ Plasmid |
| **7** | CPV-js1 | KJ754512 | Jiangsu | 2013 | **positive** | CPV-2a | Establish method |
| **8** | CPV-js2 | KJ754513 | Jiangsu | 2013 | **positive** | CPV-2b | Establish method |
| **9** | CPV-js3 | KJ754514 | Jiangsu | 2013 | **positive** | CPV-2a | Establish method |
| **10** | CPV-js4 | KJ754515 | Jiangsu | 2013 | **positive** | CPV-2c | Establish method |
| **11** | CPV-js5 | KJ754516 | Jiangsu | 2013 | **positive** | CPV-2a | Establish method |
| **12** | CPV-js6 | KJ754517 | Jiangsu | 2013 | **positive** | CPV-2a | Establish method |
| **13** | CPV-js7 | KJ754518 | Jiangsu | 2013 | **positive** | CPV-2b | Establish method |
| **14** | CPV-js8 | KJ754519 | Jiangsu | 2013 | **positive** | CPV-2a | Establish method |
| **15** | CPV-js9 | KJ754520 | Jiangsu | 2013 | **positive** | CPV-2a | Establish method |
| **16** | CPV-js10 | KJ754521 | Jiangsu | 2013 | **positive** | CPV-2a | Establish method |
| **17** | CPV-js11/ CPV-js11-2 | KJ754522/ KJ754523 | Jiangsu | 2013 | **positive** | CPV-2a/2b | Establish method |
| **18** | CPV-js12 | KJ754524 | Jiangsu | 2013 | **positive** | CPV-2a | Establish method |
| **19** | CPV-js13 | KJ754525 | Jiangsu | 2013 | **positive** | CPV-2a | Establish method |
| **20** | CPV-js14 | KJ754526 | Jiangsu | 2013 | **positive** | CPV-2c | Establish method |
| **21** | CPV-js15 | KJ754527 | Jiangsu | 2013 | **positive** | CPV-2a | Establish method |
| **22** | CPV-js16 | KJ754528 | Jiangsu | 2013 | **positive** | CPV-2a | Establish method |
| **23** | CPV-js17 | KJ754529 | Jiangsu | 2013 | **positive** | CPV-2a | Establish method |
| **24** | CPV-js18 | KJ754530 | Jiangsu | 2013 | **positive** | CPV-2a | Establish method |
| **25** | CPV-js19 | KJ754531 | Jiangsu | 2013 | **positive** | CPV-2b | Establish method |
| **26** | CPV-js20 | KJ754532 | Jiangsu | 2013 | **positive** | CPV-2c | Establish method |
| **27** | CPV-js21 | KJ754533 | Jiangsu | 2013 | **positive** | CPV-2b | Establish method |
| **28** | CPV-js22 | KJ754534 | Jiangsu | 2013 | **positive** | CPV-2b | Establish method |
| **29** | CPV-js23 | KJ754535 | Jiangsu | 2013 | **positive** | CPV-2a | Establish method |
| **30** | CPV-js24 | KJ753536 | Jiangsu | 2013 | **positive** | CPV-2a | Establish method |
| **31** | CPV-js25 | KJ754537 | Jiangsu | 2013 | **positive** | CPV-2a | Establish method |
| **32** | CPV-js26 | KJ754538 | Jiangsu | 2013 | **positive** | CPV-2a | Establish method |
| **33** | CPV-js27 | KJ754539 | Jiangsu | 2013 | **positive** | CPV-2a | Establish method |
| **34** | COV-js28 | KJ754540 | Jiangsu | 2013 | **positive** | CPV-2b | Establish method |
| **35** | CPV-js29 | KJ754541 | Jiangsu | 2013 | **positive** | CPV-2a | Establish method |
| **36** | CPV-js30 | KJ754542 | Jiangsu | 2013 | **positive** | CPV-2b | Establish method |
| **37** | CPV-js31 | MK076889 | Jiangsu | 2017 | **positive** | CPV-2a | Field sample |
| **38** | CPV-js32 | MK076890 | Jiangsu | 2017 | **positive** | CPV-2a | Field sample |
| **39** | CPV-js33 | MK076891 | Jiangsu | 2017 | **positive** | CPV-2b | Field sample |
| **40** | CPV-js34 | MK076892 | Jiangsu | 2017 | **positive** | CPV-2a | Field sample |
| **41** | CPV-js35 | MK076893 | Jiangsu | 2017 | **positive** | CPV-2c | Field sample |
| **42** | CPV-js36 | MK076894 | Jiangsu | 2017 | **positive** | CPV-2b | Field sample |
| **43** | CPV-js37 | MK076895 | Jiangsu | 2017 | **positive** | CPV-2a | Field sample |
| **44** | CPV-js38 | MK076896 | Jiangsu | 2017 | **positive** | CPV-2a | Field sample |
| **45** | CPV-js39 | MK076897 | Jiangsu | 2017 | **positive** | CPV-2a | Field sample |
| **46** | CPV-js40 | MK076898 | Jiangsu | 2017 | **positive** | CPV-2a | Field sample |
| **47** | CPV-js41 | MK076899 | Jiangsu | 2017 | **positive** | CPV-2a | Field sample |
| **48** | CPV-js42 | MK076900 | Jiangsu | 2017 | **positive** | CPV-2c | Field sample |
| **49** | CPV-js43 | MK076901 | Jiangsu | 2017 | **positive** | CPV-2b | Field sample |
| **50** | CPV-js44 | MK076902 | Jiangsu | 2017 | **positive** | CPV-2b | Field sample |
| **51** | CPV-js45 | MK076903 | Jiangsu | 2017 | **positive** | CPV-2a | Field sample |
| **52** | CPV-js46 | MK076904 | Jiangsu | 2017 | **positive** | CPV-2c | Field sample |
| **53** | CPV-js47 | MK076905 | Jiangsu | 2017 | **positive** | CPV-2a | Field sample |
| **54** | CPV-js48 | MK076906 | Jiangsu | 2017 | **positive** | CPV-2a | Field sample |
| **55** | CPV-js49 | MK076907 | Jiangsu | 2017 | **positive** | CPV-2a | Field sample |
| **56** | CPV-js50 | MK076908 | Jiangsu | 2017 | **positive** | CPV-2a | Field sample |
| **57** | CPV-js51 | MK076909 | Jiangsu | 2017 | **positive** | CPV-2a | Field sample |
| **58** | CPV-js52 | MK076910 | Jiangsu | 2017 | **positive** | CPV-2a | Field sample |
| **59** | CPV-js53 | MK076911 | Jiangsu | 2017 | **positive** | CPV-2a | Field sample |
| **60** | CPV-js54 | MK076912 | Jiangsu | 2017 | **positive** | CPV-2a | Field sample |
| **61** | CPV-js55 | MK076913 | Jiangsu | 2017 | **positive** | CPV-2a | Field sample |
| **62** | CPV-js56 | MK076914 | Jiangsu | 2017 | **positive** | CPV-2a | Field sample |
| **63** | CPV-js57 | MK076915 | Jiangsu | 2017 | **positive** | CPV-2a | Field sample |
| **64** | CPV-js58 | MK076916 | Jiangsu | 2017 | **positive** | CPV-2a | Field sample |
| **65** | CPV-js59 | MK076917 | Jiangsu | 2017 | **positive** | CPV-2c | Field sample |
| **66** | CPV-js60 | MK076918 | Jiangsu | 2017 | **positive** | CPV-2a | Field sample |
| **67** | CPV-js61 | MK076919 | Jiangsu | 2017 | **positive** | CPV-2a | Field sample |
| **68** | CPV-js62 | MK076920 | Jiangsu | 2017 | **positive** | CPV-2b | Field sample |
| **69** | CPV-js63 | MK076921 | Jiangsu | 2017 | **positive** | CPV-2a | Field sample |
| **70** | CPV-js64 | MK076922 | Jiangsu | 2017 | **positive** | CPV-2a | Field sample |
| **71** | CPV-js65 | MK076923 | Jiangsu | 2017 | **positive** | CPV-2b | Field sample |
| **72** | CPV-js66 | MK076924 | Jiangsu | 2017 | **positive** | CPV-2a | Field sample |
| **73** | CPV-js67 | MK076925 | Jiangsu | 2017 | **positive** | CPV-2a | Field sample |
| **74** | CPV-js68 | MK076926 | Jiangsu | 2017 | **positive** | CPV-2b | Field sample |
| **75** | CPV-js69 | MK076927 | Jiangsu | 2017 | **positive** | CPV-2a | Field sample |
| **76** | CPV-js70 | MK076928 | Jiangsu | 2017 | **positive** | CPV-2a | Field sample |
| **77** | CPV-js71 | MK076929 | Jiangsu | 2017 | **positive** | CPV-2a | Field sample |
| **78** | CPV-js72 | MK076930 | Jiangsu | 2017 | **positive** | CPV-2a | Field sample |
| **79** | CPV-js73 | MK076931 | Jiangsu | 2017 | **positive** | CPV-2b | Field sample |
| **80** | CPV-js74 | MK076932 | Jiangsu | 2017 | **positive** | CPV-2a | Field sample |
| **81** | CPV-js75 | MK076933 | Jiangsu | 2017 | **positive** | CPV-2c | Field sample |
| **82** | CPV-js76 | MK076934 | Jiangsu | 2017 | **positive** | CPV-2a | Field sample |
| **83** | CPV-js77 | MK076935 | Jiangsu | 2017 | **positive** | CPV-2c | Field sample |
| **84** | CPV-js78 | MK076936 | Jiangsu | 2017 | **positive** | CPV-2a | Field sample |
| **85** | CPV-js79 | MK076937 | Jiangsu | 2017 | **positive** | CPV-2a | Field sample |
| **86** | CPV-js81 | MK076938 | Jiangsu | 2017 | **positive** | CPV-2a | Field sample |
| **87** | CPV-js82 | MK076939 | Jiangsu | 2017 | **positive** | CPV-2a | Field sample |
| **88** | CPV-js83 | MK076940 | Jiangsu | 2017 | **positive** | CPV-2a | Field sample |
| **89** | CPV-js84 | MK076941 | Jiangsu | 2017 | **positive** | CPV-2a | Field sample |
| **90** | CPV-js85 | MK076942 | Jiangsu | 2017 | **positive** | CPV-2a | Field sample |
| **91** | CPV-js86 | MK076943 | Jiangsu | 2017 | **positive** | CPV-2a | Field sample |
| **92** | JS19-1 | MK460553 | Jiangsu | 2019 | **positive** | CPV-2c | Field sample |
| **93** | JS19-2 | MK460554 | Jiangsu | 2019 | **positive** | CPV-2c | Field sample |
| **94** | JS19-3 | — | Jiangsu | 2019 | **negtive** | **negtive** | Field sample |
| **95** | JS19-4 | MK460555 | Jiangsu | 2019 | **negtive** | CPV-2c | Field sample |
| **96** | JS19-5 | MK460556 | Jiangsu | 2019 | **positive** | CPV-2c | Field sample |
| **97** | JS19-6 | — | Jiangsu | 2019 | **negtive** | **negtive** | Field sample |
| **98** | JS19-7 | — | Jiangsu | 2019 | **negtive** | **negtive** | Field sample |
| **99** | JS19-8 | — | Jiangsu | 2019 | **negtive** | **negtive** | Field sample |
| **100** | JS19-9 | — | Jiangsu | 2019 | **negtive** | **negtive** | Field sample |
| **101** | JS19-10 | MK460557 | Jiangsu | 2019 | **negtive** | CPV-2c | Field sample |
| **102** | JS19-11 | — | Jiangsu | 2019 | **negtive** | **negtive** | Field sample |
| **103** | JS19-12 | — | Jiangsu | 2019 | **negtive** | **negtive** | Field sample |
| **104** | JS19-13 | — | Jiangsu | 2019 | **negtive** | **negtive** | Field sample |
| **105** | JS19-14 | — | Jiangsu | 2019 | **negtive** | **negtive** | Field sample |
| **106** | JS19-15 | MK460558 | Jiangsu | 2019 | **negtive** | CPV-2c | Field sample |
| **107** | JS19-16 | — | Jiangsu | 2019 | **negtive** | **negtive** | Field sample |
| **108** | JS19-17 | — | Jiangsu | 2019 | **negtive** | **negtive** | Field sample |
| **109** | JS19-18 | — | Jiangsu | 2019 | **negtive** | **negtive** | Field sample |
| **110** | JS19-19 | MK460559 | Jiangsu | 2019 | **positive** | CPV-2c | Field sample |
| **111** | JS19-20 | MK460560 | Jiangsu | 2019 | **negtive** | CPV-2c | Field sample |
| **112** | JS19-21 | — | Jiangsu | 2019 | **negtive** | **negtive** | Field sample |
| **113** | JS19-22 | — | Jiangsu | 2019 | **negtive** | **negtive** | Field sample |
| **114** | JS19-23 | — | Jiangsu | 2019 | **negtive** | **negtive** | Field sample |
| **115** | JS19-24 | — | Jiangsu | 2019 | **negtive** | **negtive** | Field sample |
| **116** | JS19-25 | — | Jiangsu | 2019 | **negtive** | **negtive** | Field sample |
| **117** | JS19-26 | — | Jiangsu | 2019 | **negtive** | **negtive** | Field sample |
| **118** | JS19-27 | — | Jiangsu | 2019 | **negtive** | **negtive** | Field sample |
| **119** | JS19-28 | — | Jiangsu | 2019 | **negtive** | **negtive** | Field sample |
| **120** | B849A02 | — | Nobivac® DHPPi | 2013 | — | CPVint | Commercial vaccine |
| **121** | A303618 | — | Vanguard® Plus5 | 2013 | — | CPVpf/original CPV-2 | Commercial vaccine |
| **122** | A412119 | — | Vanguard® Plus5 | 2014 | — | CPVpf/original CPV-2 | Commercial vaccine |
| **123** | 176809A | — | Vanguard® Plus5 | 2016 | — | CPVpf/original CPV-2 | Commercial vaccine |

Notes:

^a^ GICT: Immune colloidal gold technique

^b^: Known genotypes of thirty samples (CPV-js1 to CPV-js30) were used to establish the method.

**Supplementary Table S2. The results of 83 clinical specimens and four CPV-2 vaccines tested using the bicolor FMCA assay**

| **Sample Number** | **Sample ID** | ***Tm* value ((°C)** | | | ***ΔTm* value (°C)** | | **Genotype** | **Interpretation** |
| --- | --- | --- | --- | --- | --- | --- | --- | --- |
|  |  | **P1(HEX channel)** | **P2(FAM channel)** | **^a^P1(HEX channel)** | | **^b^P2(FAM channel)** |  |  |
| **1** | p-CPV-2 | **55.08** | 58.37 | 0 | | 0.12 | original CPV-2 | Reference/Plasmid |
| **2** | pCPV-2a | 49.68 | **58.25** | 5.4 | | 0 | CPV-2a | Reference/Plasmid |
| **3** | pCPV-2b | 49.99 | 51.91 | 5.09 | | 6.34 | CPV-2b | Control/ Plasmid |
| **4** | pCPV-2c | 49.55 | 48.44 | 5.53 | | 9.81 | CPV-2c | Control/ Plasmid |
| **5** | pCPVpf | 55.01 | 52.28 | 0.07 | | 5.97 | CPVpf^e^ | Control/ Plasmid |
| **6** | pCPVint | 50.45 | 58.23 | 4.63 | | 0.02 | CPVint^f^ | Control/ Plasmid |
| **7** | CPV-js31 | 49.44 | 58 | 5.64 | | 0.25 | CPV-2a | Field sample |
| **8** | CPV-js32 | 49.46 | 58.01 | 5.62 | | 0.24 | CPV-2a | Field sample |
| **9** | CPV-js33 | 49.65 | 51.56 | 5.43 | | 6.69 | CPV-2b | Field sample |
| **10** | CPV-js34 | 49.64 | 58.15 | 5.44 | | 0.1 | CPV-2a | Field sample |
| **11** | CPV-js35 | 49.55 | 48.45 | 5.53 | | 9.8 | CPV-2c | Field sample |
| **12** | CPV-js36 | 49.83 | 51.92 | 5.25 | | 6.33 | CPV-2b | Field sample |
| **13** | CPV-js37 | 49.92 | 58.51 | 5.16 | | 0.26 | CPV-2a | Field sample |
| **14** | CPV-js38 | 50.17 | 58.59 | 4.91 | | 0.34 | CPV-2a | Field sample |
| **15** | CPV-js39 | 49.78 | 58.25 | 5.3 | | 0 | CPV-2a | Field sample |
| **16** | CPV-js40 | 49.51 | 58.43 | 5.57 | | 0.18 | CPV-2a | Field sample |
| **17** | CPV-js41 | 49.67 | 58.19 | 5.41 | | 0.06 | CPV-2a | Field sample |
| **18** | CPV-js42 | 49.43 | 48.32 | 5.65 | | 9.93 | CPV-2c | Field sample |
| **19** | CPV-js43 | 49.66 | 51.56 | 5.42 | | 6.69 | CPV-2b | Field sample |
| **20** | CPV-js44 | 49.39 | 51.51 | 5.69 | | 6.74 | CPV-2b | Field sample |
| **21** | CPV-js45 | 49.63 | 58.14 | 5.45 | | 0.11 | CPV-2a | Field sample |
| **22** | CPV-js46 | 49.43 | 48.53 | 5.65 | | 9.72 | CPV-2c | Field sample |
| **23** | CPV-js47 | 49.57 | 58.23 | 5.51 | | 0.02 | CPV-2a | Field sample |
| **24** | CPV-js48 | 49.72 | 58.15 | 5.36 | | 0.1 | CPV-2a | Field sample |
| **25** | CPV-js49 | 50.24 | 58.65 | 4.84 | | 0.4 | CPV-2a | Field sample |
| **26** | CPV-js50 | 49.86 | 58.51 | 5.22 | | 0.26 | CPV-2a | Field sample |
| **27** | CPV-js51 | 50.09 | 58.72 | 4.99 | | 0.47 | CPV-2a | Field sample |
| **28** | CPV-js52 | 49.68 | 58.19 | 5.4 | | 0.06 | CPV-2a | Field sample |
| **29** | CPV-js53 | 49.63 | 58.17 | 5.45 | | 0.08 | CPV-2a | Field sample |
| **30** | CPV-js54 | 49.36 | 58.12 | 5.72 | | 0.13 | CPV-2a | Field sample |
| **31** | CPV-js55 | 49.32 | 57.9 | 5.76 | | 0.35 | CPV-2a | Field sample |
| **32** | CPV-js56 | 49.33 | 58.09 | 5.75 | | 0.16 | CPV-2a | Field sample |
| **33** | CPV-js57 | 50.16 | 58.69 | 4.92 | | 0.44 | CPV-2a | Field sample |
| **34** | CPV-js58 | 49.97 | 58.49 | 5.11 | | 0.24 | CPV-2a | Field sample |
| **35** | CPV-js59 | 49.49 | 48.39 | 5.59 | | 9.86 | CPV-2c | Field sample |
| **36** | CPV-js60 | 49.65 | 58.11 | 5.43 | | 0.14 | CPV-2a | Field sample |
| **37** | CPV-js61 | 49.79 | 58.44 | 5.29 | | 0.19 | CPV-2a | Field sample |
| **38** | CPV-js62 | 49.76 | 51.66 | 5.32 | | 6.59 | CPV-2b | Field sample |
| **39** | CPV-js63 | 49.65 | 58.18 | 5.43 | | 0.07 | CPV-2a | Field sample |
| **40** | CPV-js64 | 49.63 | 58.17 | 5.45 | | 0.08 | CPV-2a | Field sample |
| **41** | CPV-js65 | 50 | 52.12 | 5.08 | | 6.13 | CPV-2b | Field sample |
| **42** | CPV-js66 | 49.32 | 57.93 | 5.76 | | 0.32 | CPV-2a | Field sample |
| **43** | CPV-js67 | 49.3 | 58.11 | 5.78 | | 0.14 | CPV-2a | Field sample |
| **44** | CPV-js68 | 49.5 | 51.42 | 5.58 | | 6.83 | CPV-2b | Field sample |
| **45** | CPV-js69 | 49.27 | 58.06 | 5.81 | | 0.19 | CPV-2a | Field sample |
| **46** | CPV-js70 | 49.47 | 58.05 | 5.61 | | 0.2 | CPV-2a | Field sample |
| **47** | CPV-js71 | 49.9 | 58.03 | 5.18 | | 0.22 | CPV-2a | Field sample |
| **48** | CPV-js72 | 49.52 | 58.04 | 5.56 | | 0.21 | CPV-2a | Field sample |
| **49** | CPV-js73 | 50.55 | 52.44 | 4.53 | | 5.81 | CPV-2b | Field sample |
| **50** | CPV-js74 | 49.76 | 58.27 | 5.32 | | 0.02 | CPV-2a | Field sample |
| **51** | CPV-js75 | 49.68 | 48.56 | 5.4 | | 9.69 | CPV-2c | Field sample |
| **52** | CPV-js76 | 49.67 | 58.21 | 5.41 | | 0.04 | CPV-2a | Field sample |
| **53** | CPV-js77 | 49.39 | 48.28 | 5.69 | | 9.97 | CPV-2c | Field sample |
| **54** | CPV-js78 | 49.34 | 57.74 | 5.74 | | 0.51 | CPV-2a | Field sample |
| **55** | CPV-js79 | 49.33 | 58.13 | 5.75 | | 0.12 | CPV-2a | Field sample |
| **56** | CPV-js81 | 50.09 | 58.68 | 4.99 | | 0.43 | CPV-2a | Field sample |
| **57** | CPV-js82 | 50.26 | 58.65 | 4.82 | | 0.4 | CPV-2a | Field sample |
| **58** | CPV-js83 | 49.44 | 57.84 | 5.64 | | 0.41 | CPV-2a | Field sample |
| **59** | CPV-js84 | 49.32 | 57.9 | 5.76 | | 0.35 | CPV-2a | Field sample |
| **60** | CPV-js85 | 49.49 | 58.02 | 5.59 | | 0.23 | CPV-2a | Field sample |
| **61** | CPV-js86 | 49.78 | 58.23 | 5.3 | | 0.02 | CPV-2a | Field sample |
| **62** | JS19-1 | 49.97 | 49.28 | ^c^ 5.23 | | ^c^ 8.84 | CPV-2c | Field sample |
| **63** | JS19-2 | 49.83 | 48.71 | ^c^ 5.37 | | ^c^ 9.41 | CPV-2c | Field sample |
| **64** | JS19-3 | — | — | — | | — | negtive | Field sample |
| **65** | JS19-4 | 49.92 | 49.24 | ^c^ 5.28 | | ^c^ 8.88 | CPV-2c | Field sample |
| **66** | JS19-5 | 49.80 | 48.90 | ^c^ 5.40 | | ^c^ 9.22 | CPV-2c | Field sample |
| **67** | JS19-6 | — | — | — | | — | negtive | Field sample |
| **68** | JS19-7 | — | — | — | | — | negtive | Field sample |
| **69** | JS19-8 | — | — | — | | — | negtive | Field sample |
| **70** | JS19-9 | — | — | — | | — | negtive | Field sample |
| **71** | JS19-10 | 49.89 | 49.36 | ^c^ 5.31 | | ^c^ 8.76 | CPV-2c | Field sample |
| **72** | JS19-11 | — | — | — | | — | negtive | Field sample |
| **73** | JS19-12 | — | — | — | | — | negtive | Field sample |
| **74** | JS19-13 | — | — | — | | — | negtive | Field sample |
| **75** | JS19-14 | — | — | — | | — | negtive | Field sample |
| **76** | JS19-15 | 49.95 | 49.43 | ^c^ 5.25 | | ^c^ 8.69 | CPV-2c | Field sample |
| **77** | JS19-16 | — | — | — | | — | negtive | Field sample |
| **78** | JS19-17 | — | — | — | | — | negtive | Field sample |
| **79** | JS19-18 | — | — | — | | — | negtive | Field sample |
| **80** | JS19-19 | 49.60 | 48.91 | ^c^ 5.60 | | ^c^ 9.21 | CPV-2c | Field sample |
| **81** | JS19-20 | 49.24 | 49.55 | ^c^ 5.96 | | ^c^ 8.57 | CPV-2c | Field sample |
| **82** | JS19-21 | — | — | — | | — | negtive | Field sample |
| **83** | JS19-22 | — | — | — | | — | negtive | Field sample |
| **84** | JS19-23 | — | — | — | | — | negtive | Field sample |
| **85** | JS19-24 | — | — | — | | — | negtive | Field sample |
| **86** | JS19-25 | — | — | — | | — | negtive | Field sample |
| **87** | JS19-26 | — | — | — | | — | negtive | Field sample |
| **88** | JS19-27 | — | — | — | | — | negtive | Field sample |
| **89** | JS19-28 | — | — | — | | — | negtive | Field sample |
| **90** | B849A02 | 50.53 | 58.25 | 4.55 | | 0 | CPVint | Commercial vaccine |
| **91** | A303618 | 54.94 | 52.45/58.61 | 0.14 | | 5.8/0.36 | CPVpf/original CPV-2 | Commercial vaccine |
| **92** | A412119 | 54.89 | 52.59/58.56 | 0.19 | | 5.66/0.31 | CPVpf/original CPV-2 | Commercial vaccine |
| **93** | 176809A | 54.91 | 52.62/58.58 | 0.17 | | 5.63/0.33 | CPVpf/original CPV-2 | Commercial vaccine |

**^a^:***ΔTm*= |*Tm* (reference control of original CPV-2) - *Tm* (other )|

**^b^:***ΔTm*= |*Tm* (reference control of CPV-2a) - *Tm* (other )|

**^c^:** Reference controls were performed in the same testing.

**Supplementary Table S3. Accuracy comparison between the FMCA and HRM methods using sequencing technique as a gold standard.**

|  | **FMCA** | **HRM^a^** | **Sequencing** |
| --- | --- | --- | --- |
| original CPV-2 | 0/113 | 0/113 | 0/113 |
| CPV-2a | 60/113 | 60/113 | 60/113 |
| CPV-2b | 15/113 | 14/113 | 15/113 |
| CPV-2c | 17/113 | 18/113 | 17/113 |
| CPV-2a/2b | 1/113 | 0/113 | 1/113 |
| NA^b^ | 0/113 | 1/113 | 0/113 |
| Accuracy | 100% | 98.23% | — |

^a^: The accuracy was calculated based on the *Tm* values from different genotypes before generating the heteroduplexes

^b^: NA = not applicable
